# Impact of rifampicin dose in bone and joint prosthetic device infections due to *Staphylococcus spp:* a retrospective single-center study in France

**DOI:** 10.1186/s12879-021-05832-2

**Published:** 2021-02-12

**Authors:** M. Tonnelier, A. Bouras, C. Joseph, Y. El Samad, B. Brunschweiler, J.-L. Schmit, C. Mabille, J-P Lanoix

**Affiliations:** 1 Infectious diseases department, CHU Amiens Nord, 1 place Victor Pauchet, 80000 Amiens, France; 2 Centre hospitalier Compiègne-Noyon – service MIPI, 8 avenue Henri Adnot, 60200 Compiègne, France; 3 UR 4294 AGIR, Université Picardie Jules Verne, 1-3 rue des Louvels, 80000 Amiens, France; 4 Orthopedic department, CHU Amiens Sud, 1 rue du Professeur Christian Cabrol, 80054 Amiens, France; 5 Pharmacy department, CHU Amiens Sud, 1 rue du Professeur Christian Cabrol, 80054 Amiens, France

**Keywords:** Prosthesis-related infections, Staphylococcus, Rifampicin, Adverse effects, Safety

## Abstract

**Background:**

Prosthetic joint infections (PJI) are a major cause of morbidity and mortality burden worldwide. While surgical management is well defined, rifampicin (RIF) dose remains controversial. The aim of our study was to determine whether Rifampicin dose impact infection outcomes in PJI due to *Staphylococcus* spp.

**Methods:**

single-center retrospective study including 411 patients with PJI due to Rifampicin-sensitive *Staphylococcus* spp. Rifampicine dose was categorized as follow: < 10 mg/kg/day, 10–20 mg/kg/day or > 20 mg/kg/day. The primary endpoint was patient recovery, defined as being free of infection during 12 months after the end of the initial antibiotic course.

**Results:**

321 (78%) received RIF for the full antibiotic course. RIF dose didn’t affect patients recovery rate with 67, 76 and 69% in the < 10, 10–20 and > 20 mg/kg/day groups, respectively (*p* = 0.083). In univariate analysis, recovery rate was significantly associated with gender (*p* = 0.012) but not to RIF dose, or *Staphylococcus* phenotype (*aureus* or coagulase-negative). In multivariate analysis, age (p = 0.01) and treatment duration (*p* <  0.01) were significantly associated with recovery rate.

**Conclusion:**

These data suggest that lower doses of RIF are as efficient and safe as the recommended high-dose French regimen in the treatment of PJI.

## Background

The disease burden of bone and joint prosthetic device infections (PJI) is high [1–3], because of a need for intensive care in 6% of cases and an estimated in-hospital lethality of 5% [4], increasing with age. Length of stay is high (between 18 and 21 days) and repeated admission rate is estimated at 19.3%, with increasing costs [1, 5, 6].

For PJI due to sensitive germs, Rifampicin (RIF) is a cornerstone, owing to its high bone and tissue diffusion and its action in biofilm [7, 8]. RIF is always used in combined therapy, most frequently with Fluoroquinolones [9–11], with an overall recommended antibiotics course duration of 6 to 12 weeks [9].

However, the ideal RIF dose for PJI is not clearly defined, varying from 5 to 20 mg/kg/day depending on the study [12–15]. The 2009 French guidelines suggested 10 mg/kg of RIF twice a day (i.e. 20 mg/kg/day) [9], whereas American guidelines (2013) did not take patient weight into account and recommended lower doses of 600 to 900 mg daily, taken once or twice (i.e. 8-12 mg/kg/day for a standard 75 kg person) [14].

Without clear data on outcomes according to RIF dose, clinicians are often guided by experiences of poor tolerance of high RIF dose [11, 16]. However, up today all pharmacokinetics studies have failed to demonstrate a correlation between RIF serum concentrations and occurrence of adverse events (AEs) [16, 17]. The rate of AEs linked to RIF in PJI varies between 4.3 and 31.2% depending on the study [12, 18–20], often attributed to variable inter-individual susceptibilities [21, 22]. Because some studies, although with low sample sizes, suggest that lower-dose RIF could remain effective while improving the drug’s tolerance [12, 20], tendency of low-dose RIF has become common without guidelines changes.

The aim of our study was to compare recovery rate in PJI due to *Staphylococcus* spp. between low-dose (< 10 mg/kg/day), intermediate dose (10-20 mg/kg/day) and high-dose (> 20 mg/kg/day) RIF. We also aimed to compare AEs occurrence in both groups.

## Methods

### Settings

This retrospective monocentric study was conducted in the Orthopedic Surgery Unit of a University Hospital in Amiens, France between January 2008 and December 2018. Our common practice in Staphylococci PJI is immediate surgery, and probabilistic IV therapy. Then after 5–7 days, oral switch to RIF plus another antibiotic is the rule. During surgery, local antimicrobials in bone cement (containing gentamicin) can be used in hip and knee infections.

Medical records were screened with the assistance of the institution’s Medical Information Department. We recorded data using the Electronic Medical Records system (EMR).

Inclusion criteria were as follows: adults over 18 years of age, hospitalized for a PJI due to RIF-sensitive *Staphylococcus* spp., treated by antibiotics regimen which included RIF for the all course of treatment.

We excluded patients with simultaneous fungal or mycobacterial infection, patients with missing data for the primary endpoint and patients with unknown RIF dose.

Clinical variables gathered included age, sex, weight, height, body-mass index (BMI), underlying comorbidities such as diabetes, active excessive alcohol consumption, immune deficiency factors (neoplasia in the current year, immunosuppressive treatment including systemic steroids (> 5 mg/kg/day), immunotherapy or chemotherapy), chronic kidney disease of grade IIIB or more and hepatic insufficiency. Surgical data included type of prosthetic device, type of surgery (one-step, two-step, arthrodesis …), infection site and microbiological data (bacterial species, number of positive samples). We classified infections as “early” or “late” when they occurred less than 3 months or more than 12 months after prosthetic device implantation, respectively [18]. We retrieved antibiotics course details including the duration (in days) and dosage of RIF, as well as the occurrence of AEs, their severity using the Common Terminology Criteria for Adverse Events (CTCAE) grades [23] and their consequences on patient management.

*Staphylococcus aureus* (SA) PJI were defined as the isolation of a SA strain (alone or not) in a surgical periprosthetic sampling. Coagulase negative *Staphylococcus* (CNS) PJI diagnosis required at least one positive intraoperative sample with compatible clinical characteristics, such as joint pain, fistula, local inflammation or presence of pus during surgery. Polymicrobial infections were defined by the co-identification of two or more bacterial species including *Staphylococcus* spp.

The primary endpoint was patient recovery, defined as being free of infection during 12 months after the end of the initial antibiotic course.

Secondary endpoints were adverse effects (AEs) and treatment failure (i.e recurrence, relapse, loss to follow-up and death). Recurrence was defined as a new PJI due to the same germ(s) as the initial infection, less than 12 months after the end of the antibiotic course. Relapse was defined as the occurrence of a new PJI due to different germ than initially, in the same interval. When 12 months clinical follow-up data were not available, patients were deemed lost to follow-up. Finally, death occurring during 12 months follow-up was considered as treatment failure.

### Statistical analysis

Quantitative variables were expressed as means and standard deviations (SD) when the distribution was normal, and median and interquartile ranges (IQR) otherwise. Normality was measured by the Skewness test. Qualitative variables were expressed as percentages. A proportion comparison test was used to analyze the qualitative variables. Fisher’s exact test was used to compare the quantitative variables. A *p*-value below 0.05 was considered significant. A logistic regression model was used to determine risk factors for failure and to account for selection bias. All statistical analyses were performed using STATA 13.1 (StataCorp, College Station, TX).

### Ethics

The study was approved by the CNIL agency (*Commission Nationale Informatique et Liberté*) in compliance with local and national regulations, under the number PI2019_843_0068.

## Results

**Fig. 1:**
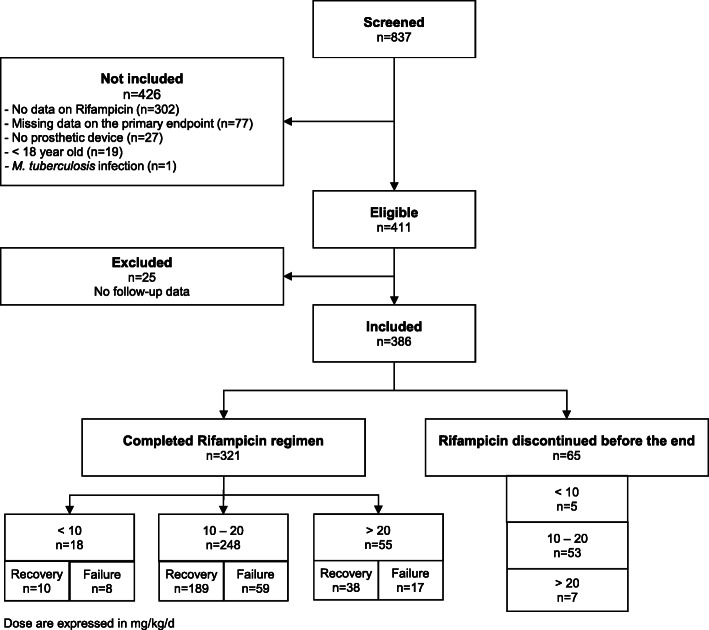
Flow chart of the study

From January 2008 until December 2018, 837 patients were treated for PJI due to *Staphylococcus* spp. (Fig. 1). A total of 411 patients were included (223 males, 54.25%) with a median age of 64.5 years (IQR [55–76], extremes 18 to 95 years), and a median weight of 82.5 kg (IQR [68–95]). (The flow chart is detailed in Fig. 1). However only 321 patients (78%) received RIF for the full length of treatment, while in 90 patients (22%) it was discontinued early because of AEs. The characteristics of the patients are detailed in Table 1.

**Table 1:** Study population characteristics

	Total	Low-dose (< 10 mg/kg/d)	Intermediate-dose (10-20 mg/kg/d)	High-dose (> 20 mg/kg/d)
**Total**, n (%)	411	27 (6.6)	319 (77.6)	65 (15.8)
**Male sex**, n (%)	223 (54.3)	9 (33.3)	181 (56.7)	33 (50.8)
**Age,** years (mean, [IQR])	64.5 [55–76]	69.2 [61–80]	64.1 [55–76]	64.6 [51–78]
**Weight**, kg (mean, [IQR])	82.5 [68–95]	99.8 [75–125]	84.5 [70–95]	65.6 [55–80]
**BMI**, kg/m^2^ (mean, [IQR])	29.1 [23. 9–33]	35.8 [27.5–42]	29.4 [24.8–33.1]	24.2 [20.4–29]
**Obesity,** n (%)	167 (40.1)	20 (74.1)	133 (41.7)	14 (21.6)
**Comorbidities**, n (%)				
Chronic alcohol consumption	34 (8.3)	2 (7.4)	26 (8.2)	6 (9.2)
Diabetes	74 (8)	7 (26)	63 (19.7)	4 (6.2)
Chronic kidney disease > grade IIIB	18 (4.4)	3 (11.1)	13 (4.1)	2 (3.1)
Immune deficiency factor	33 (10.5)	1 (3.7)	26 (8.2)	6 (9.2)
Chronic liver disease	6 (1.5)	1 (3.7)	4 (1.3)	1 (1.5)
**Infection site**, n (%)				
Hip	164 (39.9)	7 (26)	131 (41.1)	26 (40)
Femur	17 (4.1)	0 (0)	10 (3.1)	7 (10.8)
Knee	123 (9.9)	13 (48.1)	94 (29.5)	16 (24.6)
Tibia	29 (7.1)	2 (7.4)	25 (7.8)	4 (6.2)
Ankle	38 (9.2)	4 (14.8)	28 (8.8)	4 (6.2)
Foot	13 (3.2)	0 (0)	11 (3.4)	2 (3.1)
Upper limb **	28 (6.6)	1 (3.7)	20 (6.3)	6 (9.2)
**Delay of PJI**				
Acute prosthesis infection (< 3 months)	196 (47.7)	13 (48.1)	147 (46.1)	36 (55.4)
Chronic prosthesis infection (> 12 months)	130 (31.6)	9 (33.3)	104 (32.6)	17 (26.2)
**Surgical method**				
Prosthesis replacement	212 (51.6)	13 (48.1)	173 (54.2)	24 (36.9)
Prosthetic device removal	78 (19.0)	2 (7.4)	59 (18.5)	15 (23.1)
Implant retention	83 (20.7)	5 (18,5)	59 (18.5)	19 (29.2)
Arthrodesis	5 (1.2)	1 (3.7)	4 (1.3)	0 (0)
Explantation	31 (7.5)	1 (3.7)	23 (7.2)	6 (9.2)
No surgery	2	0	1	1
**Germs**, n (%)				
***Staphylococcus aureus***	289 (70.3)	19 (70.4)	229 (71.8)	42 (64.6)
Methicillin-sensitive	235 (81.3)	18 (94.7)	187 (81.7)	33 (78.6)
Methicillin-resistant	51 (17.7)	1 (5.3)	42 (18.39	9 (21.4)
**Coagulase negative** ***Staphylococcus***	122 (29.7)	8 (29.6)	90 (28.2)	23 (35.4)
Methicillin-sensitive	67 (54.9)	3 (37.5)	51 (56.7)	12 (52.2)
Methicillin-resistant	55 (40.6)	5 (62.5)	39 (43.3)	11 (47.8)
**Polymicrobial infection**	107 (26)	4 (14.8)	87 (27.3)	16 (24.6)
**Delay between prosthesis implantation and infection diagnosis,** n (%)				
< 3 months	196 (47.7)	13 (48.1)	147 (46.1)	36 (55.4)
3–12 months	85 (20.7)	5 (18.5	68 (21.3)	12 (18.5)
> 12 months	130 (31.6)	9 (33.3)	104 (32.6)	17 (26.2)
**Rifampicin treatment**				
Dosage, mg/kg/day (mean,[IQR])	15.7 [12.8–18]	8.7 [8–9.8]	14.8 [12.9–16.7]	22.9 [20.7–24]
Full treatment course, n (%)	321 (78.1)	18 (66,6)	55 (84)	248 (77,7)
**Combination treatment used**				
Fluoroquinolones	278 (67.6)	20 (74.1)	214 (67.4)	44 (67.7)
Clindamycin	64 (15.6)	5 (18.5)	51 (16)	8 (12.3)
Glycopeptides	36 (8.7)	0 (0)	28 (8.8)	8 (12.3)
Cotrimoxazole	17 (4.1)	1 (3.7)	13 (4.1)	3 (4.6)
Penicillin	6 (1.5)	0 (0)	6 (1.9)	0 (0)
Cephalosporins	2 (0.49)	1 (3.7)	1 (0.3)	0 (0)
Doxycyclin	2 (0.49)	0 (0)	2 (0.6)	0 (0)
Daptomycin	2 (0.49)	0 (0)	1 (0.3)	1 (1.5)
Oxazolidinone	2 (0.49)	0 (0)	1 (0.3)	1 (1.5)
Fosfomycin	1 (0.24)	0 (0)	1 (0.3)	0 (0)
Dalbavancin	1 (0.24)	0 (0)	1 (0.3)	0 (0)

* One or more criteria among the following: active neoplasia dating less than 1 year; immunosuppressive treatment including systemic steroids, chemotherapy or immunomodulatory drugs

** Shoulder, humerus, elbow, forearm, hand

Recovery rate was not statistically different between different groups of RIF dose: 55,6%, 76,2 and 67.3% in low-dose, intermediate-dose and high-dose groups respectively (*p* = 0.083) (Table 2). Results were similar while grouping low and intermediate dose-groups: the recovery rate was 74.8% in the < 20 mg/kg/day group and 67.3% in the > 20 mg/kg/day group. This was true when including patients who discontinued RIF before the end of treatment: 62.6% of recovery rate in the < 20 mg/kg/day group, and 62.9% in the > 20 mg/kg/day group (*p* = 0.310).

**Table 2:** Outcome for patients treated with Rifampicin for the full length of treatment (*n* = 321/411)

	Total	Low-dose	Intermediate-dose	High-dose
**Recovery**, n (%)	237 (73.8)	10 (55.5)	189 (76.2)	38 (69)
**Treatment failure**, n (%)	52 (16.2)	5 (27.8)	35 (14.1)	12 (21.8)
**Lost to follow-up**, n (%)	32 (10)	3 (16.7)	24 (9.7)	5 (9.1)

In a univariate analysis, being a female was the only variable associated with a lower recovery rate (*p* = 0.012), no association was found between recovery and RIF dosage or *Staphylococcus* phenotype (SA or CNS) or the number of germs on intraoperative sampling (Table 3). In a multivariate analysis, older age (p = 0.01) and shorter treatment duration (< 60 days, *p* <  0.01) were significantly associated with treatment failure (Table 4).

**Table 3:** Prognostic factors associated with treatment failure (univariate analysis)

Variables	Recovery	Failure	p
**Number of germs identified**			
≤ 1	178 (43.3)	126 (30.7)	0.655
> 1	64 (15.6)	43 (10.5)	
**Sex**, n (%)			
Female	99 (24.1)	89 (21.6)	0.012
Male	143 (34.8)	80 (19.6)	
**Rifampicin dosage**			
< 10 mg/kg/day	11 (2.7)	16 (3.9)	0.149
10–20 mg/kg/day	192 (46.7)	127 (30.9)	
> 20 mg/kg/day	39 (9.5)	26 (6.3)

**Table 4:** Prognostic factors associated with treatment failure (multivariate analysis)

	OR [95% CI]	p
**Age**	0,94 [0.91–0.97]	0.001
**Sex**	0.86 [0.30–2.44]	0.773
**Rifampicin dosage**, mg/kg/day	1,02 [0.9–1.15]	0.753
**Treatment duration**, days	1,05 [1.03–1.07]	< 0.001

Seventy percent of PJI were due to *SA* (*n* = 289) with 82.4% of Meticillin-sensitive *SA* (MSSA), while only 54.9% (*n* = 67) of CNS were Meticillin-sensitive. The most frequently identified CNS species was *Staphylococcus epidermidis* (67.8%). Infections were polymicrobial in 26% of cases (*n* = 107). Recovery rate was not statistically different according to staphylococcal resistance profile or in polymicrobial infections.

Median RIF dosage was 15.7 mg/kg/day (IQR [8–18], extremes 5.8–41 mg/kg/day) and only 27 patients (6.6%) received low-dose RIF (< 10 mg/kg/day). The median duration of the antibiotic course was 39 days (IQR [9–60]). RIF was mainly combined with fluoroquinolones (67.6%), clindamycin (15.6%) or glycopeptides (8.7%).

AEs attributable to RIF were reported in 106 patients (26%), leading to treatment suspension in 65 (61.3% of AEs), with a median treatment duration of 12.5 days at occurrence (IQR [5–21]). Dosage was discreased in 2 patients, and galenic was changed in 6 patients In 31 cases (29.2% of AEs), no modification was done despite middle AEs. The most frequent AEs were digestive (*n* = 51), hepatic (*n* = 12) and cutaneous (*n* = 11), usually benign with grades I – II in 75 cases (70.8% of AEs). Fifteen patients presented with grade III – IV, mainly digestive (*n* = 10) AEs. AE occurrence rate was 20.9% in the medium-dose group versus 5.2% in the high-dose group (*p* = 0.640), and 1.3% in the low-dose group (Table 5). Neither obesity (BMI > 30) nor comorbidities were a risk factor of AEs.

**Table 5:** Safety analysis

	Total	Low-dose	Intermediate-dose	High-dose
**AE occurrence**, n (%)				
Yes	106 (25.8)	5 (18.5)	81 (25.4)	20 (30.8)
No	281 (68.4)	18 (66.7)	220 (69)	43 (66.2)
Unknown	24 (5.8)	4 (14.8)	18 (5.6)	2 (3.1)
**Type of AE**, n (%)				
Digestive	51 (48.1)	2 (40)	36 (44.4)	12 (60)
Hepatic	12 (11.3)	2 (40)	11 (13.6)	0 (0)
Cutaneous	11 (10.4)	0 (0)	8 (9.9)	2 (10)
Renal	3 (2.8)	0 (0)	2 (2.5)	1 (5)
Hematological	2 (1.9)	0 (0)	2 (2.5)	0 (0)
General	1 (0,94)	0 (0)	1 (1,2)	0 (0)
Neurologic	1 (0,94)	0 (0)	1 (1,2)	0 (0)
> 1 AE types	23 (21,8)	1 (20)	17 (20,9)	4 (20)
Not specified	1 (0,94)	0 (0)	1 (1,2)	1 (5)
Unknown	1 (0,94)	0 (0)	2 (2,5)	0 (0)
**AE severity grade**, n (%)				
I – II	75 (70.8)	3 (60)	53 (65.4)	16 (80)
III – IV	15 (14.2)	2 (40)	10 (12.3)	2 (10)
Unknown	16 (15)	0 (0)	18 (22.2)	2 (10)
**AE consequences on patient management**				
Antibiotic change	62 (58.5)	5 (100)	50 (61.7)	7 (35)
Rifampicin continuation	32 (30.2)	0 (0)	20 (24.7)	11 (55)
Galenic change	6 (5.7)	0 (0)	4 (4.9)	2 (10)
Dosage decrease	2 (1.9)	0 (0)	2 (2.5)	0 (0)
Antibiotic discontinuation	4 (3.8)	0 (0)	4 (4.9)	0 (0)
Unknown	0 (0)	0 (0)	1 (1.2)	0 (0)

## Discussion

In our study, there was no difference in recovery rate between low-dose and high-dose RIF containing regimens for PJI due to *Staphylococcus* spp., both in patients who continued treatment for the recommended duration (*p* = 0.083) and in those who discontinued RIF early (*p* = 0.31). These findings are in line with previously published data in favor of using lower doses of RIF, without risking treatment failure [12, 20]. However, the absence of statistical difference could be attributed to the low power of our study (1-β = 0.221, α risk = 0.05), despite our relatively large sample size.

Treatment failure was significantly associated with duration of RIF treatment (< 60 days), but not with the dose. This finding is consistent with DATIPO study results, which found a higher risk of treatment failure with shorter courses of antibiotics (< 45 days) [24]. Another randomized multi-center clinical trial also demonstrated the non-inferiority of a combined 8-week fluoroquinolone-RIF regimen, versus 3 or 6 months for hip or knee prostheses respectively, using debridement and implant retention [25].

The rate of AEs attributable to RIF was higher in our study population compared to previously published series, in which the percentage varies from 2 to 15% [10, 13, 26, 27]. One could blame our relatively high RIF dose compared to these studies where doses ranged from 600 mg [28–30] to 900 mg daily [26, 31] regardless of patient weight. However AE occurrence was the highest in the 10–20 mg/kg/day group and the lowest in the > 20 mg/kg/day (5.2%).Several studies have similarly reported that high dose of RIF (> 30/mg/kg/day) could be used safely over a long period for the treatment of tuberculosis [32, 33]. Conversely, Hagihara et al. suggested that the toxicity associated with prolonged combined antibiotics therapy could be limited by lowering RIF dose [34] Valour et al. found a 6.5% AE rate for a median RIF dose of 18.8 mg/kg/day, with a smaller study population (*n* = 107) [18]. On the other hand, Randuineau et al. more recently suggested a two-fold increase in AEs in patients treated with RIF at > 12 versus < 12 mg/kg/day, leading to more frequent treatment discontinuation but without any significant reduction in recovery [20].

The poor tolerance of RIF could thus be solely attributable to inter-individual susceptibilities. Yet, multiple studies failed to show any influence of sex, age, height or weight on pharmacokinetic parameters of RIF [22, 35, 36]. Furthermore, studies by Roblot et al. and Dupouey et al. did not demonstrate any association between RIF serum concentrations and the occurrence of AEs neither digestive nor else [16, 17]. Besides, RIF usage should not be limited to non-comorbid patients, given the safety data of this study and previous ones [37]. While the value of pharmacological monitoring during RIF containing regimen remains unproven pharmacogenetics studies could be of help in the future [17]. Biological and clinical surveillance remain the pillar of PJI follow-up to prevent a potential harmful RIF discontinuation due to the use of less powerful antibiotics [11, 19, 27, 38–40].

In addition, we did not find lower recovery rates in patients with methicillin-resistant Staphylococci, in line with previous studies [28, 41, 42], or in patients with polymicrobial infections, in contrast to a study by Senneville et al. [19].

This study has some limitations. Missing data are inherent to the retrospective nature of our work, cutting off 102 patients from our initial sample. The low number of patients receiving low-dose RIF has probably contributed to our low study power.

## Conclusion

In conclusion, our data show the feasibility of using lower doses of RIF than these currently advised by national guidelines, which is consistent with the results of several observational studies [12, 20, 28, 29, 31]. A national French randomized controlled trial (PHRC EVRIOS – NTC 02599493), programmed for 2021, aims to include 460 patients, with the goal of determining optimal RIF dosage (< 10 or > 20 mg/kg/day) in PJI due to *Staphylococcus* spp., and will probably definitely answer this question*.*

## Data Availability

The datasets used and/or analysed during the current study are available from the corresponding author on reasonable request.

## Abbreviations

AE: Adverse effects
CTCAE: Common Terminology Criteria for Adverse Events
EMR: Electronic Medical Records
IQR: Interquartile ranges
PJI: Joint prosthetic device infections
RIF: Rifampicin
SA: *Staphylococcus aureus*
SD: Standard deviations
